# Reversible autoinhibition of the bacterial replication-licensing Hda–β clamp complex

**DOI:** 10.1093/nar/gkag1010

**Published:** 2026-10-23

**Authors:** Xuguang Jiang, Radostin Danev, Yanbin Luan, Makoto Nakakido, Kouhei Tsumoto, Masahide Kikkawa

**Affiliations:** Department of Cell Biology and Anatomy, Graduate School of Medicine, The University of Tokyo, Bunkyo, Tokyo 113-0033, Japan; Department of Cell Biology and Anatomy, Graduate School of Medicine, The University of Tokyo, Bunkyo, Tokyo 113-0033, Japan; Department of Cell Biology and Anatomy, Graduate School of Medicine, The University of Tokyo, Bunkyo, Tokyo 113-0033, Japan; Department of Bioengineering, School of Engineering, The University of Tokyo, Bunkyo, Tokyo 113-8656, Japan; Department of Chemistry and Biotechnology, School of Engineering, The University of Tokyo, Bunkyo, Tokyo 113-8656, Japan; Department of Bioengineering, School of Engineering, The University of Tokyo, Bunkyo, Tokyo 113-8656, Japan; Department of Chemistry and Biotechnology, School of Engineering, The University of Tokyo, Bunkyo, Tokyo 113-8656, Japan; Medical Device Development and Regulation Research Center, School of Engineering, The University of Tokyo, Bunkyo, Tokyo 113-8655, Japan; The Institute of Medical Science, The University of Tokyo, Bunkyo, Tokyo 108-8639, Japan; Department of Cell Biology and Anatomy, Graduate School of Medicine, The University of Tokyo, Bunkyo, Tokyo 113-0033, Japan

## Abstract

Bacterial DNA replication must be tightly controlled to ensure once-per-cell-cycle initiation, requiring precise regulation of the initiator DnaA. The conserved RIDA pathway inactivates DnaA through the Hda–β sliding clamp (DnaN) complex, yet how this system is dynamically regulated has remained unclear. Here, we show that the Hda–DnaN complex exists as a concentration-dependent oligomeric ensemble rather than a static assembly. Using cryo-electron microscopy and mass photometry, we resolve multiple structural states, including an autoinhibited hetero-octamer, a hetero-hexamer intermediate, and a RIDA-compatible hetero-trimer. Structural comparison of these states suggests a plausible route of oligomeric interconversion involving progressive destabilization of Hda self-association, accompanied by exposure of the catalytic arginine finger required for DnaA inactivation. We further propose that this equilibrium operates as a titration-like mechanism that converts local protein concentration into functional states. Together, our findings establish reversible oligomerization as a potential regulatory principle for bacterial replication licensing.

## Graphical Abstract

**Graphical Abstract:**
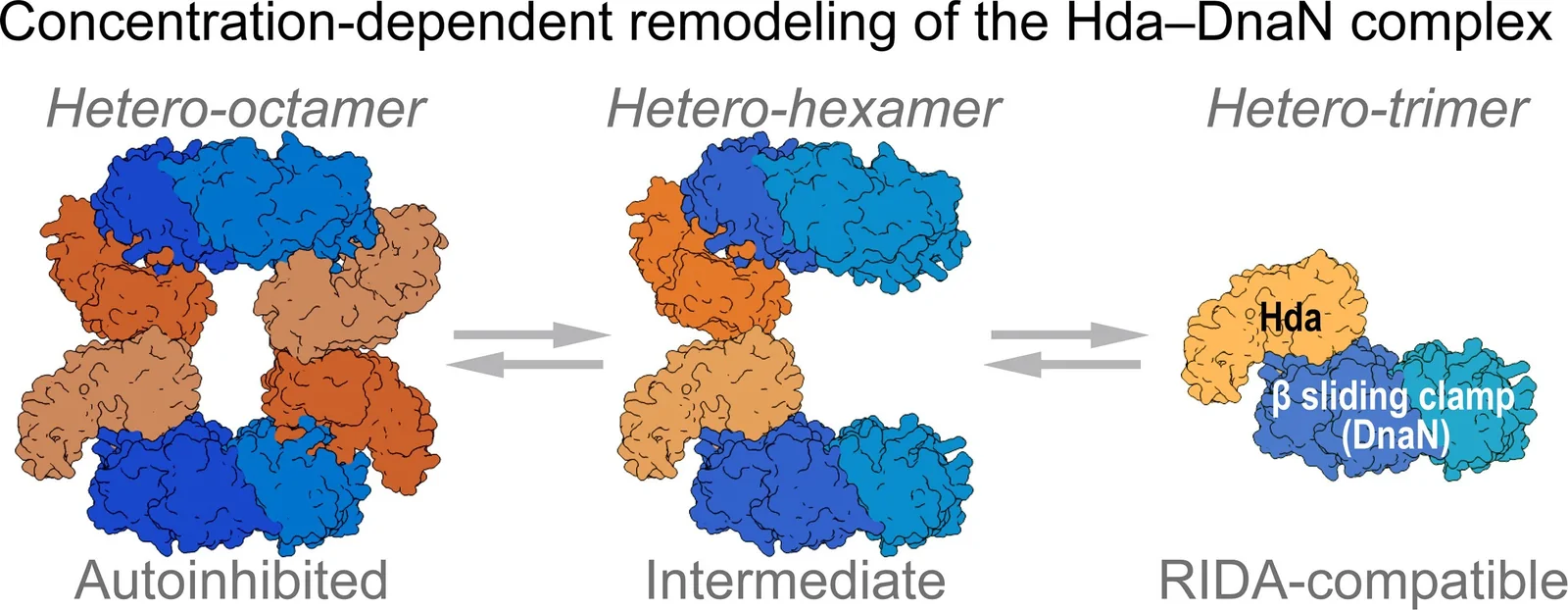

## Introduction

DNA replication must be initiated only once per cell cycle to preserve genome integrity across all domains of life. In bacteria, this process is orchestrated by the replication initiator DnaA, whose ATP-bound form assembles at the chromosomal origin to melt origin DNA and recruit the replisome [1–4]. Premature, delayed, or repeated replication initiation causes severe fitness defects, replication stress, and genome instability, making initiation control one of the most fundamental problems in chromosome biology [5–7].

A central hub in bacterial DNA replication is the DNA polymerase processivity factor β sliding clamp (DnaN), a ring-shaped clamp that encircles DNA and tethers multiple enzymes to the replication fork [8, 9]. Beyond its canonical role in polymerase processivity, DnaN acts as a dynamic signaling platform that coordinates DNA replication, Okazaki fragment maturation, DNA repair, and checkpoint-like regulatory pathways through sequential interactions with diverse partner proteins [8, 10–15]. Thus, DnaN is increasingly recognized not merely as a passive processivity factor, but as a multifunctional organizer of replication-cycle transitions [13, 16, 17].

One of the most conserved bacterial mechanisms that prevents re-initiation is Regulatory Inactivation of DnaA (RIDA), in which Hda (homologous to DnaA), a DnaA-related AAA+ ATPase family protein, is recruited to the DNA-loaded DnaN and stimulates ATP hydrolysis on DnaA, converting active ATP–DnaA into inactive ADP–DnaA. RIDA couples replication initiation control to replication fork progression, thereby ensuring that new rounds of initiation are suppressed once DNA synthesis has begun [7]. Genetic and biochemical studies have established RIDA as a major licensing pathway in *Escherichia* and related bacteria, functioning together with additional systems such as datA- and DARS-mediated nucleotide-state control to generate robust oscillations in DnaA activity during the cell cycle [1, 2, 18, 19].

However, how the Hda–DnaN complex itself is regulated has remained unclear. A landmark crystal structure showed that Hda and DnaN assemble into an inactive hetero-octameric complex in which the catalytic arginine finger of Hda is buried at the oligomeric interface, suggesting an autoinhibited storage state [20]. Disrupting this architecture compromises RIDA and causes replication defects *in vivo*. Yet this structure also raised a central mechanistic question: how can an apparently stable self-inhibited oligomer access lower-order configurations compatible with RIDA activity? Whether Hda–DnaN exists as a static octamer or as a reversible ensemble of interconverting states has remained unknown.

More broadly, recent work has revived the concept that bacterial replication timing emerges from coupled homeostatic systems involving DnaA activation, titration on chromosomal binding sites, and fork-dependent feedback control [5, 21, 22]. In this framework, protein assemblies that sense replication status and reversibly modulate DnaA activity are expected to play outsized regulatory roles. However, structural evidence for such dynamic regulatory assemblies has been scarce.

Here, we combine cryo-electron microscopy (cryo-EM) and mass photometry (MP) to reveal that the Hda–DnaN complex is not a static, inactive particle, but a concentration-dependent dynamic ensemble that samples multiple oligomeric states. We determine cryo-EM structures of an autoinhibited hetero-octamer (Hda_4_–DnaN_4_), a previously unknown hetero-hexamer intermediate (Hda_2_–DnaN_4_), an active hetero-trimer (Hda_1_–DnaN_2_), and the free DnaN dimer. Together with molecular dynamics (MD) and AlphaFold modeling, these data provide a structural framework for reversible Hda–DnaN oligomerization that links Hda self-association to the accessibility of its DnaA-interacting surface.

## Materials and methods

### Constructs, protein expression, and purification

The gene encoding DnaN from *Escherichia coli* (NCBI accession: NP_418156.1) was amplified by polymerase chain reaction and cloned into a pET-28a vector containing an N-terminal hexahistidine tag. The gene encoding Hda (NCBI accession: NP_416991.2) was cloned into a pET-21b vector without an affinity tag.

The two plasmids were co-transformed into *E. coli* BL21 (DE3) cells (Novagen), and transformants were selected using both ampicillin and kanamycin. A single colony was cultured in LB medium at 37°C until the optical density at 600 nm reached 0.6–0.8, followed by induction with 0.3 mM isopropyl β-D-1-thiogalactopyranoside at 16°C for 20 h.

Cells were harvested by centrifugation and lysed by sonication. The lysate was clarified by centrifugation at 15 000 × *g* for 30 min, and the supernatant was used for purification.

For affinity purification, the supernatant was applied to a Ni–NTA agarose column (Fujifilm) equilibrated with buffer containing 20 mM Tris–HCl (pH 7.5), 500 mM NaCl, and 5% (v/v) glycerol. Bound proteins were eluted with buffer supplemented with 400 mM imidazole. The eluate was further purified by size-exclusion chromatography (SEC) using a Superdex 200 Increase column (Cytiva) equilibrated in 20 mM HEPES-KOH (pH 7.5), 150 mM KCl, 1 mM dithiothreitol, 1 mM ethylene glycol-bis(β-aminoethyl ether)-N,N,N′,N′-tetraacetic acid (EGTA), and 2 mM MgCl_2_.

Purified proteins were concentrated to ∼10 mg/ml using ultrafiltration, flash-frozen in liquid nitrogen, and stored at −80°C.

### Mass photometry analysis

MP measurements were performed using a TwoMP instrument (Refeyn) at 25°C following established protocols [23, 24]. Samples were prepared in buffer containing 10 mM HEPES-KOH (pH 7.4), 150 mM KCl, 1 mM EGTA, and 2 mM MgCl_2_.

For measurements in the absence of DNA, Hda–DnaN samples at concentrations ranging from 0.625 to 20 nM (from SEC peak 1, Fig. 1c) and 20 nM His-DnaN (peak 2, Fig. 1c) were analyzed.

**Figure 1.**
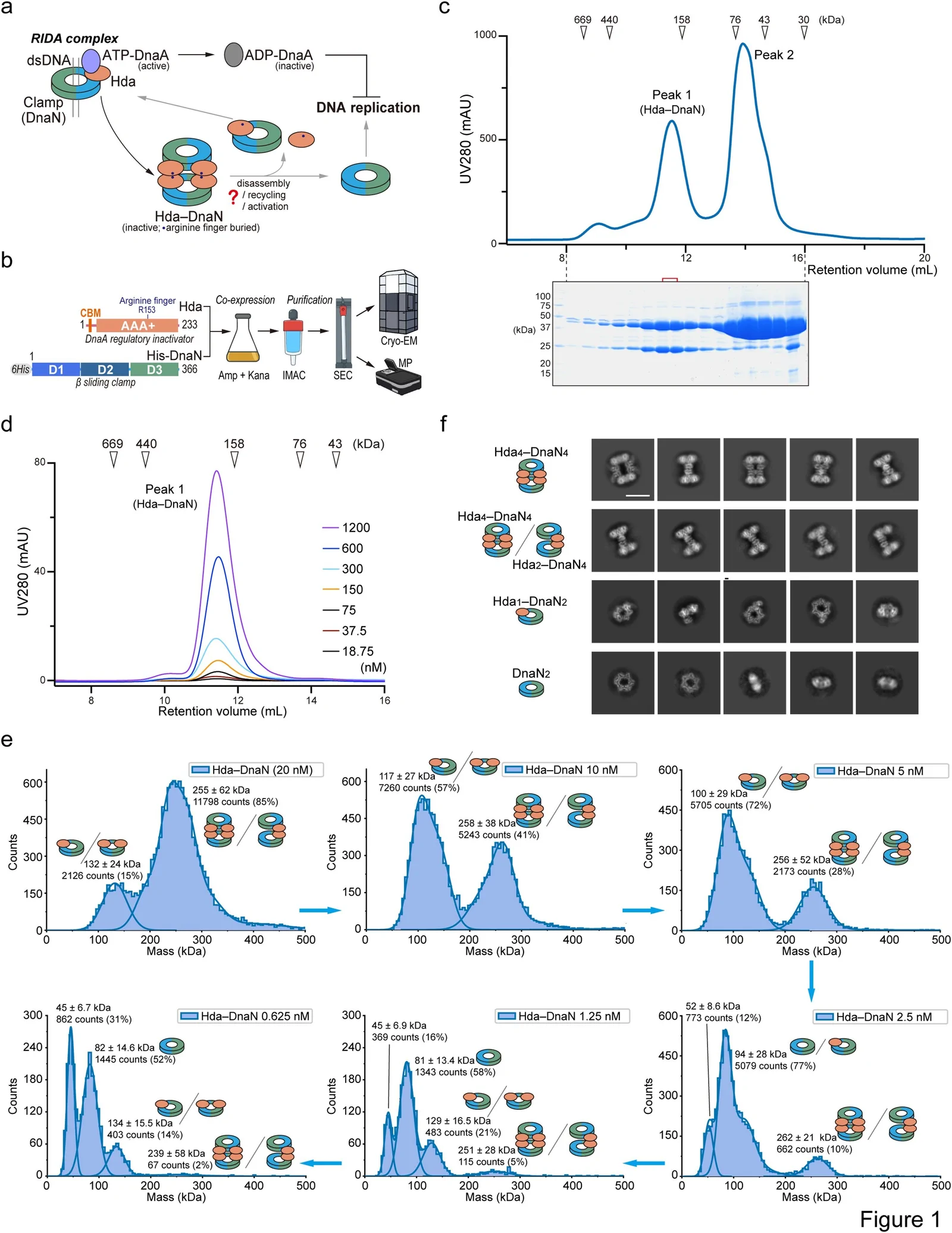
Identification and concentration-dependent oligomerization of multiple Hda–DnaN assemblies. (**A**) Schematic illustration of RIDA. Hda is recruited to DnaN via a clamp-binding motif (CBM) and subsequently engages ATP-bound DnaA to form the RIDA complex, promoting ATP hydrolysis and converting DnaA into its inactive ADP-bound form, thereby preventing replication initiation. In the absence of substrate, Hda–DnaN can assemble into an inactive hetero-octamer (Hda_4_–DnaN_4_), in which the arginine finger (Arg153) is buried within the interface. Disruption of this assembly has been reported to impair RIDA and lead to defects in the bacterial cell cycle. However, how this inactive complex disassembles and recycles into the active state remains unclear. (**B**) Domain organization of Hda and DnaN constructs and schematic of the experimental workflow. Co-expression of Hda and His-tagged DnaN was followed by purification and subsequent cryo-EM and MP analyses. CBM, clamp-binding motif; D1–D3, domains 1–3 of DnaN; IMAC, immobilized metal affinity chromatography; SEC, size-exclusion chromatography. (**C**) SEC profile of the co-expressed Hda–DnaN sample after IMAC and corresponding sodium dodecyl sulfate–polyacrylamide gel electrophoresis analysis. Two major peaks are observed, corresponding to the Hda–DnaN complex (peak 1) and excess His-DnaN. (**D**) Concentration-gradient SEC analysis of the Hda–DnaN complex (SEC peak 1). (**E**) MP analysis of the Hda–DnaN complex under different concentrations. Measured molecular weights and particle counts are shown, together with the corresponding stoichiometric assignments. (**F**) Cryo-EM 2D classification and representative class averages of the Hda–DnaN complex, revealing multiple coexisting assemblies, including the hetero-octamer (Hda_4_–DnaN_4_), hetero-hexamer (Hda_2_–DnaN_4_), hetero-trimer (Hda_1_–DnaN_2_), and DnaN dimer (DnaN_2_). Scale bar, 10 nm.

For measurements in the presence of DNA, complementary oligonucleotides were synthesized for a 16-bp duplex (5′-GAGATAGTTACAACAT-3′) and a 40-bp duplex (5′-GCGATCGTACGTGAGATAGTTACAACATACGTACGATCGC-3′). Equimolar complementary strands were annealed by heating to 95°C, followed by gradual cooling to room temperature. Hda–DnaN (10 nM) was incubated with 50 nM double-stranded DNA (dsDNA) for 30 min before MP measurement.

Molecular weights of the samples were calculated using DiscoverMP software (Refeyn). Representative MP distributions are shown in Figs 1e and 6a. Molecular-weight statistics for the concentration-dependent measurements shown in Fig. 1e are summarized in Table 1, and the complete MP distributions, including mirrored background events, are provided in Supplementary Fig. S2. Complete distributions from the replicate measurements performed in the absence or presence of 16- or 40-bp dsDNA are provided in Supplementary Fig. S5.

**Table 1.** MP statistics for concentration-dependent Hda–DnaN measurements and the DnaN-only control

Protein	Concentration (nM)	MW of peaks (kDa)	Counts (proportion)
Hda–DnaN	20	255 ± 62	11 798 (85%)
		132 ± 24	2126 (15%)
	10	258 ± 38	5243 (41%)
		117 ± 27	7260 (57%)
	5	256 ± 52	2173 (28%)
		100 ± 29	5705 (72%)
	2.5	262 ± 21	662 (10%)
		94 ± 28	5079 (77%)
		52 ± 8.6	773 (12%)
	1.25	251 ± 28	115 (5%)
		129 ± 16.5	483 (21%)
		81 ± 13.4	1343 (58%)
		45 ± 6.9	369 (16%)
	0.625	239 ± 58	67 (2%)
		134 ± 15.5	403 (14%)
		82 ± 14.6	1445 (52%)
		45 ± 6.7	862 (31%)
His-DnaN	20	85 ± 23	12 521 (100%)

### Cryo-EM sample preparation and data collection

Three microliters of the Hda–DnaN complex at 1.5 mg/ml (∼6 μM) was applied to glow-discharged holey carbon grids (Cu, R1.2/1.3, 300 mesh, Quantifoil). Grids were blotted for 4 s (blot force 10) and plunge-frozen in liquid ethane using a Vitrobot Mark IV (FEI) at 6°C and 100% humidity.

Data were collected on a CRYO ARM 200 (JEOL) transmission electron microscope operating at 200 kV, using SerialEM for automated acquisition [25]. Movies were recorded on a Gatan K3 direct electron detector in counting mode with correlated double sampling, at a nominal magnification of 80 000×, corresponding to a calibrated pixel size of 0.571 Å.

Data were acquired in zero-loss mode with an energy filter slit width of 20 eV and an objective aperture of 150 μm. A total of 12 396 movies were collected with a total exposure of 76.4 e⁻/Å² over 80 frames. The target defocus was set for −1.0 to −1.3 μm (measured range −0.4 to −1.8 μm). Imaging parameters are summarized in Table 2.

**Table 2.** Cryo-EM data collection, modeling, and refinement statistics

	Hda_4_–DnaN_4_	Hda_2_–DnaN_4_	Hda_1_–DnaN_2_	DnaN_2_
**Data collection and processing**				
Microscopy	CRYO ARM 200			
Detector	Gatan K3			
Voltage	200 kV			
Cs (mm)	1.55			
Slit width (eV)	20			
Defocus range (μm)	0.4–1.8			
Electron exposure (e/Å^2^)	76.4			
Pixel size (Å)	0.571			
Fractions (no.)	80			
Movies collected/used	12 396/12 206			
Single particles (no.)	9 437 582			
Single particles used (no.)	292 612	321 931	445 610	89 946
Overall resolution (Å)	3.12	3.43	3.17	3.55
FSC threshold	0.143			
**Model building and refinement**				
Model composition				
Non-hydrogen atoms	18 587	12 946	7308	4781
Protein residues	2384	1786	962	732
Ligands (Mg-ADP)	4	2	1	0
R.m.s. deviation				
Bond lengths (Å)	0.003	0.004	0.004	0.005
Bond angles (°)	0.636	1.113	0.821	1.186
Validation				
MolProbity score	1.52	1.88	1.55	1.77
Clashscore	9	13	11	13
Poor rotamers (%)	0	0	0	0
Ramachandran plot				
Favored (%)	97.76	96.05	98.43	97.25
Allowed (%)	2.24	3.95	1.57	2.75
Outliers (%)	0	0	0	0
**PDB**	25RY	25RZ	25SA	25WZ
**EMDB**	EMD-80 334	EMD-80 335	EMD-80 336	EMD-80 433

### Cryo-EM single-particle data processing

All image processing was carried out using cryoSPARC (version 5.0.2) [26].

Raw movies were initially processed using cryoSPARC Live, including patch motion correction to compensate for beam-induced motion and patch CTF estimation to determine contrast transfer function parameters. Dose-weighted micrographs were exported for downstream analysis. Micrographs with a CTF fit worse than 8 Å were discarded, yielding 12 206 high-quality images.

Initial particle picking was performed using a blob picker to generate templates, followed by iterative 2D classification. Refined templates were then used for template-based picking. To improve angular coverage and account for particle size heterogeneity, multiple rounds of picking were performed using different particle diameters.

Particles were extracted with 4× binning (pixel size 2.284 Å) and subjected to several rounds of 2D classification using masks of different sizes (110–150 Å). Selected particles were merged and deduplicated, resulting in a dataset of 9 437 582 particles.

A subset of particles was used for *ab initio* reconstruction to generate initial 3D models, which were further classified using heterogeneous refinement. Selected classes were re-extracted at 2× binning (pixel size 1.142 Å) and subjected to additional rounds of *ab initio* reconstruction and heterogeneous refinement to remove low-quality particles. Four well-defined 3D classes were obtained, corresponding to Hda_4_–DnaN_4_ hetero-octamer, Hda_2_–DnaN_4_ hetero-hexamer, Hda_1_–DnaN_2_ hetero-trimer, and DnaN_2_ homodimer, respectively.

Due to projection similarities and shared subunits among these assemblies, particle misclassification was observed. To address this, each class was further refined through iterative 2D classification and 3D classification (with and without alignment) to remove misassigned particles.

Final particle sets were refined using homogeneous or non-uniform refinement with CTF refinement and local motion correction. Local resolution was estimated, and maps were sharpened using DeepEMhancer [27] or local filtering. The processing workflow is shown in Supplementary Fig. S3, and statistics are summarized in Table 2.

### Atomic model building and validation

Initial atomic models were generated by docking. For the Hda_4_–DnaN_4_ complex, the crystal structure (PDB: 5 × 06) was docked into the cryo-EM map using UCSF ChimeraX. For the Hda_2_–DnaN_4_ and Hda_1_–DnaN_2_ complexes, truncated heterotrimeric models derived from the crystal structure were docked using Phenix [28], with two or one copies, respectively. For the DnaN_2_ dimer, an AlphaFold 3-predicted model was docked manually.

Models were refined using real-space refinement in Phenix, followed by manual adjustment in Coot [29]. Structural validation was performed using MolProbity [30]. Refinement statistics are summarized in Table 2.

### Analytical SEC assays

SEC experiments were performed using a Superdex 200 10/300 column (Cytiva) on an ÄKTA Explorer system as previously described [31]. Samples (500 μl) at seven concentrations (1200 to 18.75 nM) were analyzed. Molecular weight standards included thyroglobulin (669 kDa), ferritin (440 kDa), aldolase (158 kDa), conalbumin (75 kDa), ovalbumin (44 kDa), and carbonic anhydrase (30 kDa). Data were analyzed using GraphPad Prism7 (RRID:SCR_002798).

### Molecular dynamics simulation and free energy calculation

MD simulations were carried out using GROMACS (version 2024.4). Missing residues were modeled using SWISS-MODEL, and systems were parameterized using the OPLS-AA force field.

Each system was solvated in a cubic box with SPC/E water and 150 mM NaCl. Energy minimization was followed by equilibration (100 ps NVT and 100 ps NPT), and production simulations were run for 1 ns.

Binding free energies were estimated using the MM/PBSA method with the gmx_MMPBSA tool [32], using the last 10 frames (0.9–1 ns). Energies were decomposed into electrostatic, van der Waals, and solvation contributions.

To simulate disassembly, one Hda dimer was removed from the Hda_4_–DnaN_4_ model, and a parallel MD simulation was performed. Interface energy changes are shown in Fig. 4d.

### AlphaFold prediction and structural analysis

Structure prediction of the DnaA–Hda–DnaN_2_ complex was performed using AlphaFold 3 [33]. Interface buried areas were calculated using PISA (RRID:SCR_015749). Structural alignment and RMSD calculations were performed using PyMOL (RRID:SCR_000305), and figures were generated using UCSF ChimeraX. Theoretical molecular weights were calculated using ProtParam (RRID:SCR_018087). Additional structures (PDB: 5FKW [34], 8BTG [35], and 8VAP [36]) were used for visualization in Fig. 6.

## Results

### The Hda–DnaN complex exists in multiple concentration-dependent oligomeric states

Physiologically, Hda is recruited to the DNA polymerase β sliding clamp DnaN via its CBM and subsequently interacts with DnaA to form the RIDA complex, thereby stimulating ATP hydrolysis and inactivating DnaA [2, 5]. The crystal structure reported by Kim *et al*. revealed that the Hda–DnaN complex predominantly adopts an inactive hetero-octameric assembly *in vitro*, in which the catalytic arginine finger (Arg153) is buried within the oligomeric interface. Disruption of this assembly *in vivo* leads to defective RIDA activity and aberrant DNA replication [20]. However, the mechanism by which this autoinhibited state is disassembled and recycled into an active form remains unclear (Fig. 1a).

We hypothesized that the Hda–DnaN complex undergoes a concentration-dependent conformational equilibrium that governs its functional transitions, even in the absence of known upstream regulators.

To test this hypothesis, we co-expressed Hda and His-tagged DnaN and purified the complex using affinity chromatography followed by SEC (Fig. 1b and c). The SEC profile revealed two major peaks corresponding to the Hda–DnaN complex (peak 1) and excess His–DnaN (peak 2) (Fig. 1c). Based on SEC molecular weight standards, peak 1 was likely consistent with the previously reported hetero-octamer [20], whereas peak 2 corresponded to the DnaN homodimer. Fractions from the center of peak 1 were collected for further analysis.

To assess whether multiple oligomeric states coexist, we performed concentration-gradient analytical SEC. However, even at concentrations approaching the detection limit (18.75 nM), only a single peak was observed (Fig. 1d), consistent with previous reports [20]. Thus, distinct oligomeric species could not be resolved by SEC under these conditions.

We therefore employed MP to probe the oligomeric distribution at equilibrium. MP analysis revealed several distinct molecular weight populations that shift toward lower-molecular-weight species upon dilution, indicating a concentration-dependent redistribution (Fig. 1e and Table 1). Based on theoretical molecular weights (MWs) (Supplementary Fig. S1a), these species were assigned to the hetero-octamer (Hda_4_–DnaN_4_) and/or hetero-hexamer (Hda_2_–DnaN_4_), hetero-trimer (Hda_1_–DnaN_2_) and/or hetero-tetramer (Hda_2_–DnaN_2_), and DnaN homodimer (DnaN_2_) (Fig. 1e). At concentrations below 2.5 nM, additional signals were observed in the ∼45–52 kDa range. However, because this mass range approaches the lower detection limit of MP and partially overlaps with mirrored background events, its molecular assignment remains uncertain (Supplementary Fig. S2). The presence of intermediate assemblies such as the hetero-hexamer (Hda_2_–DnaN_4_) or hetero-tetramer cannot be excluded due to peak broadening.

In contrast, MP analysis of peak 2 revealed a dominant ∼85-kDa species corresponding to the stable DnaN dimer (Supplementary Fig. S1b).

To further validate these assignments, we performed cryo-EM single-particle analysis. 2D classification revealed distinct particle classes corresponding to Hda_4_–DnaN_4_, Hda_2_–DnaN_4_, Hda_1_–DnaN_2_, and DnaN_2_ (Fig. 1f).

Due to technical requirements for cryo-EM grid preparation, the sample concentration used for cryo-EM analysis (1.5 mg/ml, ∼6 μM) was substantially higher than those used for SEC and MP measurements. Based on the SEC and MP profiles, the biochemical behavior at this concentration would be expected to be dominated by the Hda_4_–DnaN_4_ hetero-octamer. Nevertheless, multiple oligomeric states were successfully identified through extensive classification (Fig. 1f), further supporting the existence of a dynamic conformational equilibrium.

Together, these results support a concentration-dependent oligomeric equilibrium of the Hda–DnaN complex involving multiple higher- and lower-order assemblies.

### Cryo-EM structures reveal a hetero-hexamer intermediate within the Hda–DnaN oligomeric landscape

The coexistence of multiple oligomeric states suggested a possible structural route connecting higher- and lower-order assemblies. To examine this possibility, we collected an extensive cryo-EM dataset and performed iterative 2D and 3D classifications (Supplementary Fig. S3, Table 2).

Despite significant compositional heterogeneity and particle misalignment/misclassification arising from shared substructures, our extensive classification and particle-cleaning procedures successfully reconstructed four distinct cryo-EM maps at resolutions of 3.12, 3.43, 3.17, and 3.55 Å, corresponding to Hda_4_–DnaN_4_, Hda_2_–DnaN_4_, Hda_1_–DnaN_2_, and DnaN_2_, respectively (Fig. 2a, Supplementary Fig. S3, and Table 2).

**Figure 2.**
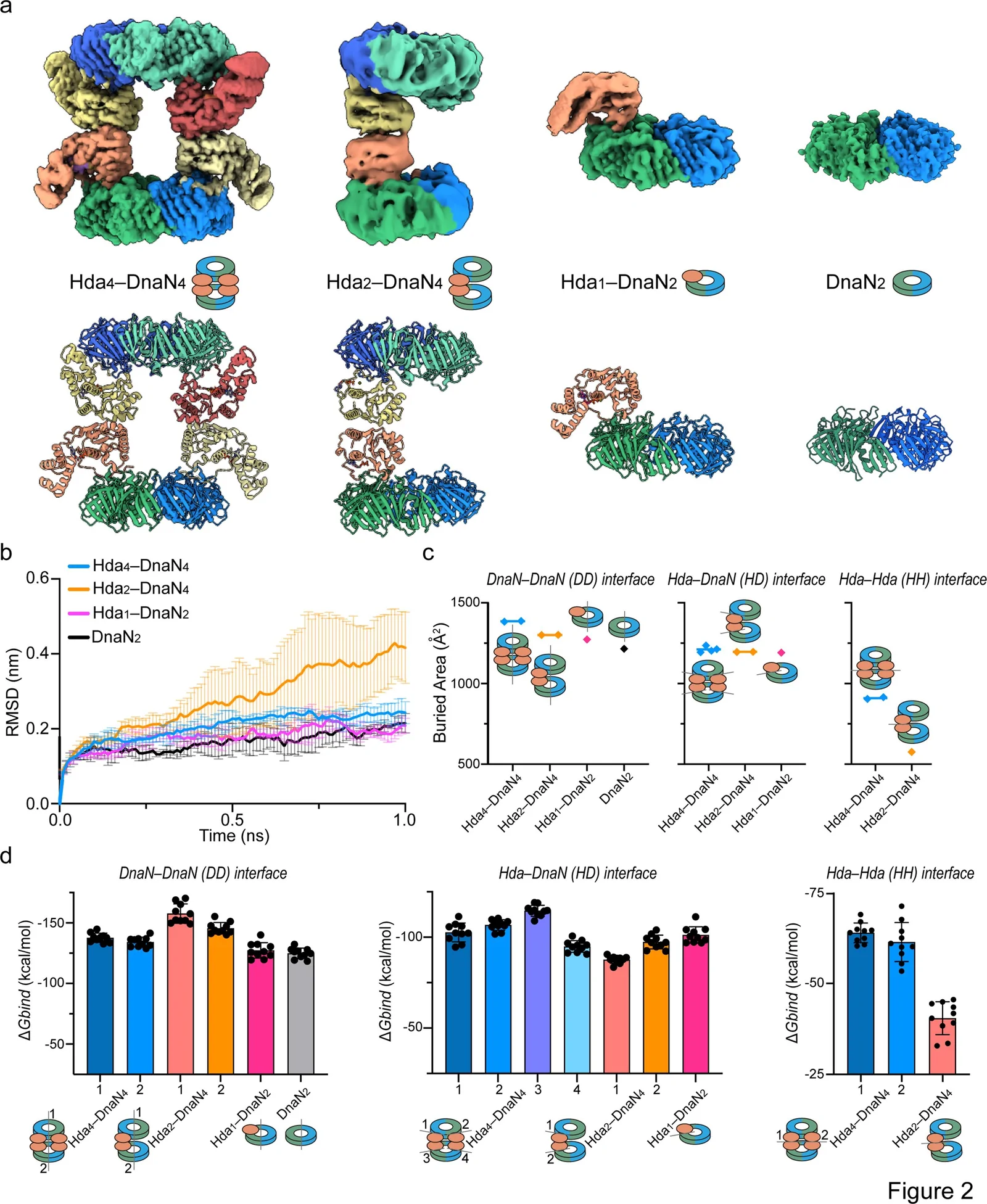
Cryo-EM reconstruction of multiple Hda–DnaN assemblies and interface analysis. (**A**) Cryo-EM single-particle reconstruction of the Hda–DnaN complex. Four distinct assemblies were resolved. Cryo-EM density maps (upper panels) and corresponding atomic models (lower panels) are shown. (**B**) MD simulations of the four assemblies. Missing regions and side chains were modeled using SWISS-MODEL and subjected to 1-ns MD simulations. The mean ± SD is plotted. (**C**) Buried surface areas of the Hda–Hda, Hda–DnaN, and DnaN–DnaN interfaces in the four assemblies. Calculations were performed with energy-minimized models with missing residues rebuilt, as determined by PISA. Mean values are shown as lines. (**D**) Binding free energy calculations based on MD-MMPBSA. The last 10 frames of each MD trajectory were used to evaluate the Hda–Hda, Hda–DnaN, and DnaN–DnaN interfaces. Bar graphs show the mean ± SD.

Importantly, these structures revealed a previously uncharacterized hetero-hexameric intermediate (Hda_2_–DnaN_4_). Although additional heterogeneous hetero-hexameric states could not be resolved due to limited particle numbers, local resolution analysis showed reduced resolution in the DnaN ring region of the hexamer, while the Hda dimer remained well resolved (Supplementary Fig. S3), suggesting increased flexibility of DnaN in the hetero-hexameric state. On the other hand, the hetero-tetramer (Hda_2_–DnaN_2_) was not observed by cryo-EM. Within the set of structures resolved here, the Hda_2_–DnaN_4_ assembly therefore provides a plausible structural connection between the higher-order Hda_4_–DnaN_4_ and lower-order Hda_1_–DnaN_2_ states, although the directionality of interconversion cannot be inferred from these static structures.

The structural comparison supports a plausible disassembly route connecting Hda_4_–DnaN_4_, Hda_2_–DnaN_4_, Hda_1_–DnaN_2_, and DnaN_2_. In this structural model, loss of one Hda dimer from the hetero-octamer would generate the hetero-hexamer, whereas subsequent weakening of the Hda–Hda interface could favor formation of lower-order Hda_1_–DnaN_2_ assemblies.

To further evaluate these transitions, we performed 1-ns MD simulations. Among the four assemblies, the hetero-hexamer exhibited the largest structural variability, consistent with greater conformational flexibility of this assembly (Fig. 2b).

Interface analysis revealed that DnaN–DnaN (DD) and Hda–DnaN (HD) interfaces remain relatively stable across states, whereas the Hda–Hda (HH) interface in the hetero-hexamer shows a marked reduction in buried surface area and binding free energy compared to the hetero-octamer (Figs 2c and d). This supports a model in which destabilization of the HH interface could facilitate formation of the hetero-trimer state.

### The Hda_4_–DnaN_4_ hetero-octamer adopts a stable autoinhibited conformation

The cryo-EM structure of the hetero-octamer exhibits clear D2 symmetry and closely matches the previously reported crystal structure [20] (RMSD ∼1.8 Å), indicating that the autoinhibited assembly is preserved in solution (Fig. 3a).

**Figure 3.**
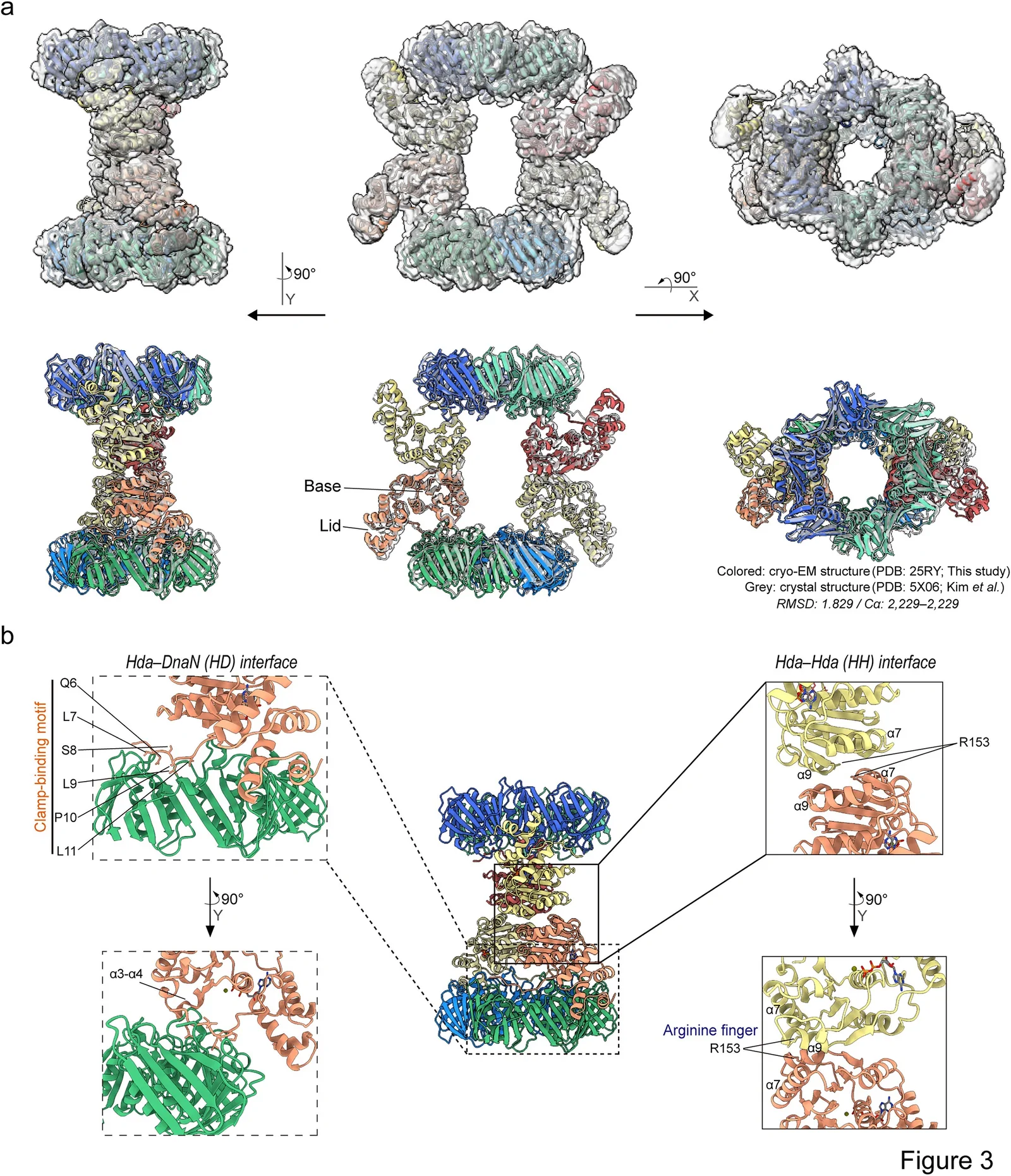
Structure and interaction interfaces of the Hda_4_–DnaN_4_ hetero-octamer. (**A**) Cryo-EM map and atomic model of the Hda_4_–DnaN_4_ complex. The model is superimposed onto the density map (transparent gray; contour level 0.01). (**B**) Structural analysis of the Hda–DnaN (left) and Hda–Hda (right) interfaces. Left: Hda engages DnaN via the CBM and helices α2–α3, interacting with hydrophobic pockets formed by domains 3 and 2 of DnaN, respectively. Right: The Hda–Hda dimer interface is primarily mediated by helices α7 and α9. The arginine finger (Arg153), located on helix α9, is buried within the dimer interface.

Both the lid and base domains of Hda are well resolved, suggesting a rigid conformation. Interface analysis shows that Hda engages DnaN via the canonical CBM and additional contacts between helices α2–α3 and DnaN domain II (Fig. 3b, left), forming a stable HD interface (∼−130 kcal/mol) (Fig. 2d). Hda binds on the front face of DnaN, following the canonical binding arrangement used by many clamp-interacting proteins, including replicative DNA polymerases.

The HH interface is mediated primarily by helices α7 and α9 (Fig. 3b, right), with a binding energy of ∼−60 kcal/mol (Fig. 2d). Notably, the catalytic Arg153 is buried within this interface, consistent with an autoinhibited state that prevents interaction with DnaA.

### The hetero-hexamer reveals structural remodeling of the Hda–Hda interface

Compared to the octamer, the hetero-hexamer displays increased heterogeneity and reduced symmetry. Reconstruction required C1 refinement, as imposing C2 symmetry, even with the symmetry relaxation, resulted in misalignment (Supplementary Fig. S3).

While most regions are well resolved, the Hda lid domain shows weak density, indicating increased flexibility (Fig. 4a). Structural alignment revealed that the two hetero-trimers remain similar (RMSD ∼1.9 Å) (Supplementary Fig. S4a), but relative orientations are altered (Fig. 4a).

**Figure 4.**
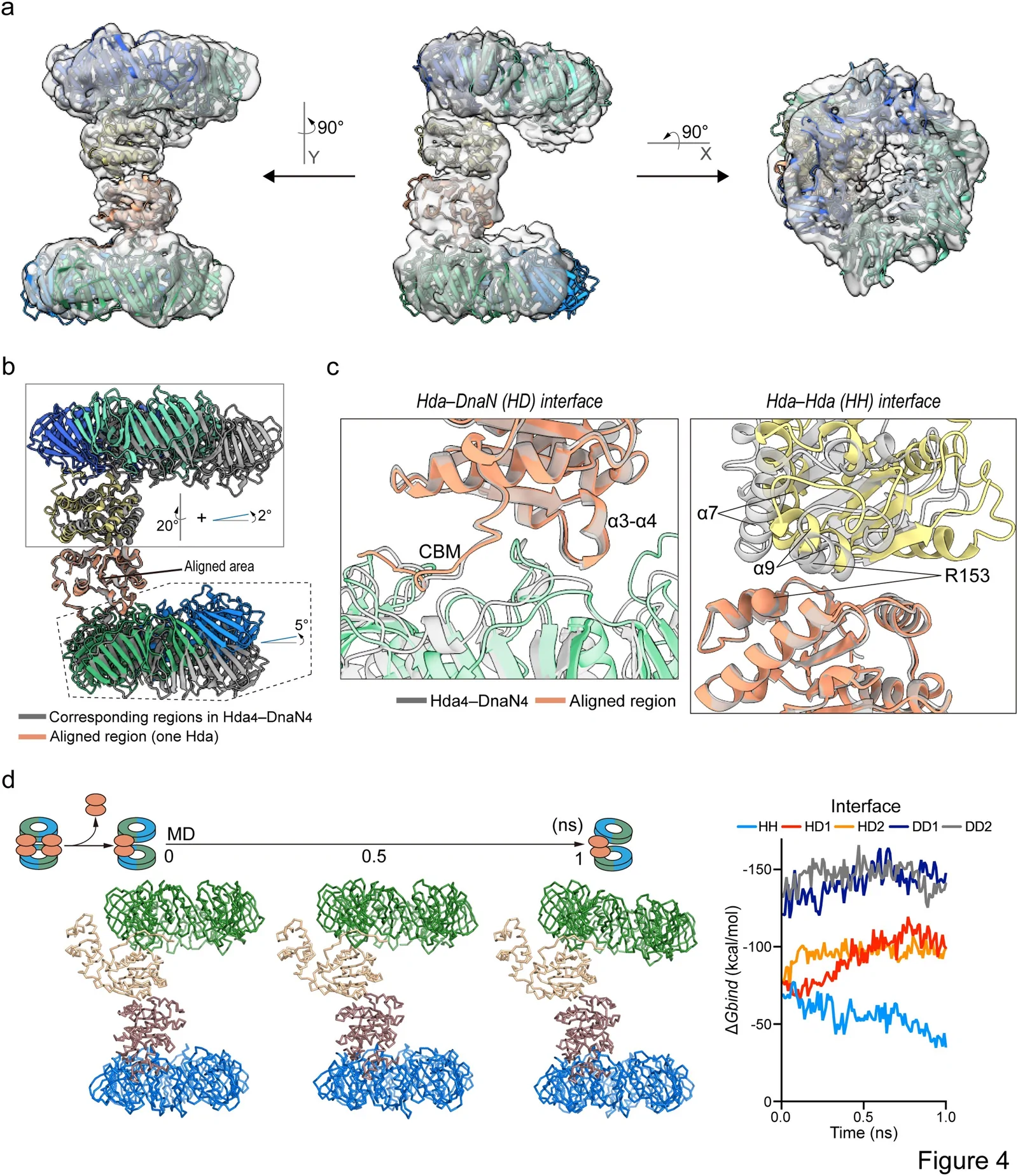
Structure of the Hda_2_–DnaN_4_ hetero-hexamer and comparison with the hetero-octamer. (**A**) Cryo-EM map and atomic model of the Hda_2_–DnaN_4_ hetero-hexamer (contour level 0.005). (**B**) Structural comparison between the Hda_2_–DnaN_4_ and Hda_4_–DnaN_4_ assemblies. Alignment was performed based on a single Hda subunit. The relative rotations of the associated DnaN dimer and the second heterotrimeric unit are indicated. (**C**) Structural alignment of the Hda–DnaN and Hda–Hda interfaces between the Hda_2_–DnaN_4_ and Hda_4_–DnaN_4_ complexes. (**D**) MD simulation of the Hda_2_–DnaN_4_ complex. The initial model was generated by removing one Hda dimer from the Hda_4_–DnaN_4_ assembly. The left panel shows backbone ribbon representations of the structure at 0, 0.5, and 1 ns during the simulation. The right panel summarizes the changes in binding free energy for each interface throughout the MD trajectory.

Using one Hda as a reference, we observed ∼5° rotation of the associated DnaN ring and ∼20° rotation of the second hetero-trimer (Fig. 4b), suggesting structural rearrangement following Hda dimer dissociation.

Our interface comparison shows that the HD interface remains largely unchanged, whereas the HH interface undergoes distortion, particularly in helices α7 and α9 (Fig. 4c), consistent with the reduced binding energy (Fig. 2d, right panel).

MD simulations further support this model: removal of one Hda dimer induces inward deformation of the DnaN ring and progressive weakening of the HH interface, while HD and DD interfaces remain stable (Fig. 4d, Movie 1).

### The lower-order hetero-trimer exposes the DnaA-interacting surface

We next analyzed the solved Hda_1_–DnaN_2_ and DnaN_2_ structures (Fig. 5a and 5b). The Hda_1_–DnaN_2_ structure closely resembles the trimeric unit in the octamer (RMSD ∼0.88 Å) and maintains a stable HD interface (Fig. 5c and Supplementary Fig. S4b). The DnaN ring is also structurally indistinguishable from the isolated DnaN_2_ dimer (Supplementary Fig. S4c). Although the DnaN construct contains an N-terminal His-tag, the observed DnaN_2_ structure adopts the canonical head-to-tail β-clamp architecture rather than a tag-mediated dimer configuration, indicating that the observed dimerization is unlikely to result from His-tag-induced self-association [37].

**Figure 5.**
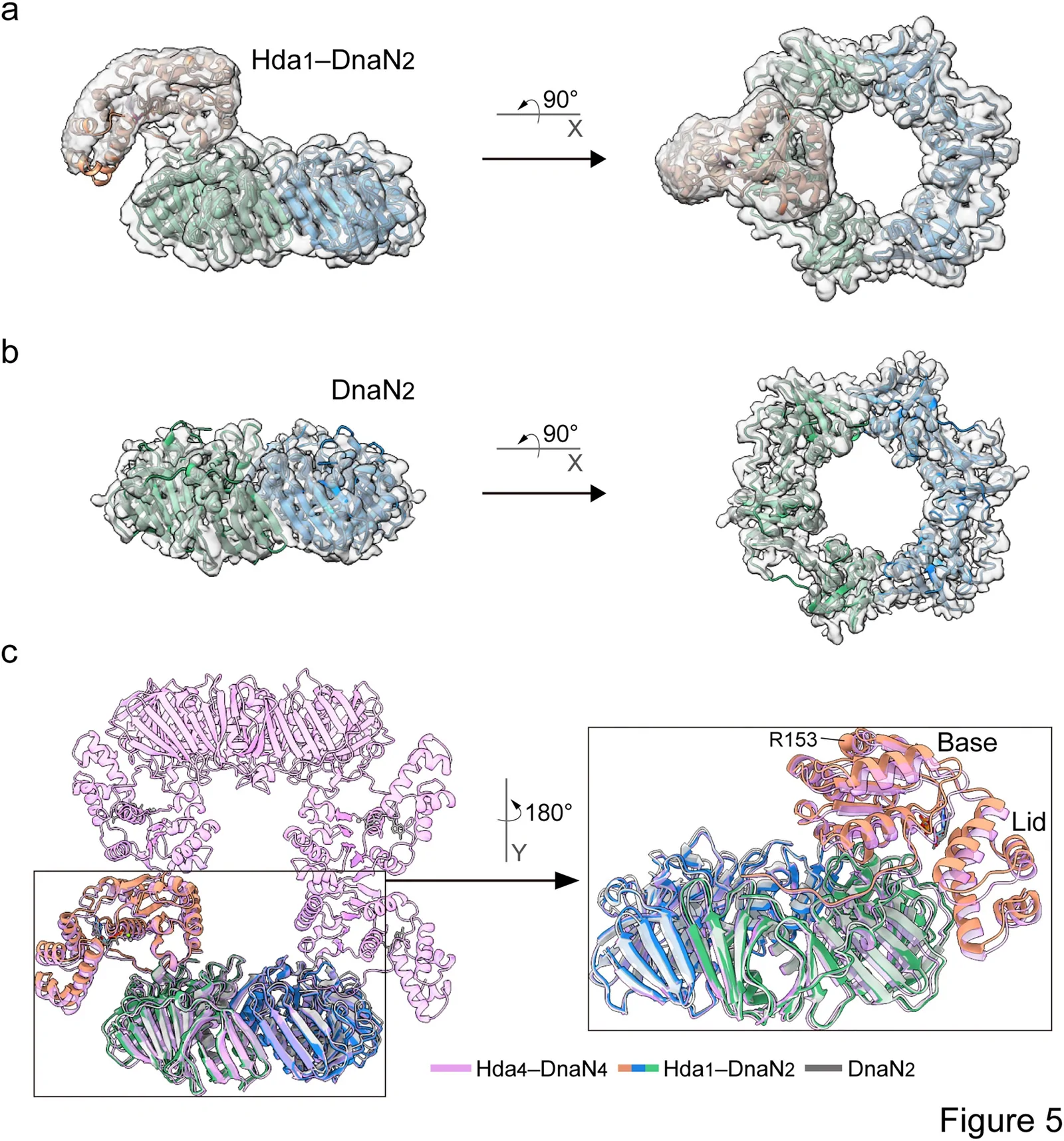
Structural comparison of the RIDA-compatible Hda_1_–DnaN_2_ complex with other oligomeric assemblies. (**A**) Cryo-EM map and model of the Hda_1_–DnaN_2_ complex (contour level 0.1). (**B**) Cryo-EM map and model of the DnaN dimer (DnaN_2_) (contour level 0.05). (**C**) Structural superposition of Hda_1_–DnaN_2_ with DnaN_2_ and Hda_4_–DnaN_4_.

Importantly, in this state, the arginine finger (Arg153) and surrounding functional surfaces are fully exposed (Fig. 5c), consistent with a RIDA-compatible conformation capable of engaging DnaA.

### Free duplex DNA does not consistently alter the Hda–DnaN oligomeric distribution

Because physiological RIDA occurs in the context of DNA-loaded DnaN, we next examined whether the presence of DNA alone influences the oligomeric distribution of the Hda–DnaN complex. We performed MP using 10 nM Hda–DnaN in the absence or presence of a fivefold molar excess (50 nM) of either 16- or 40-bp dsDNA (Fig. 6a and Supplementary Fig. S5). The 16-bp duplex was designed based on the short duplex DNA substrates used in previous structural studies of the *E. coli* clamp-loading system [15], and the 40-bp sequence was obtained by further extending both ends of the 16-bp DNA.

**Figure 6.**
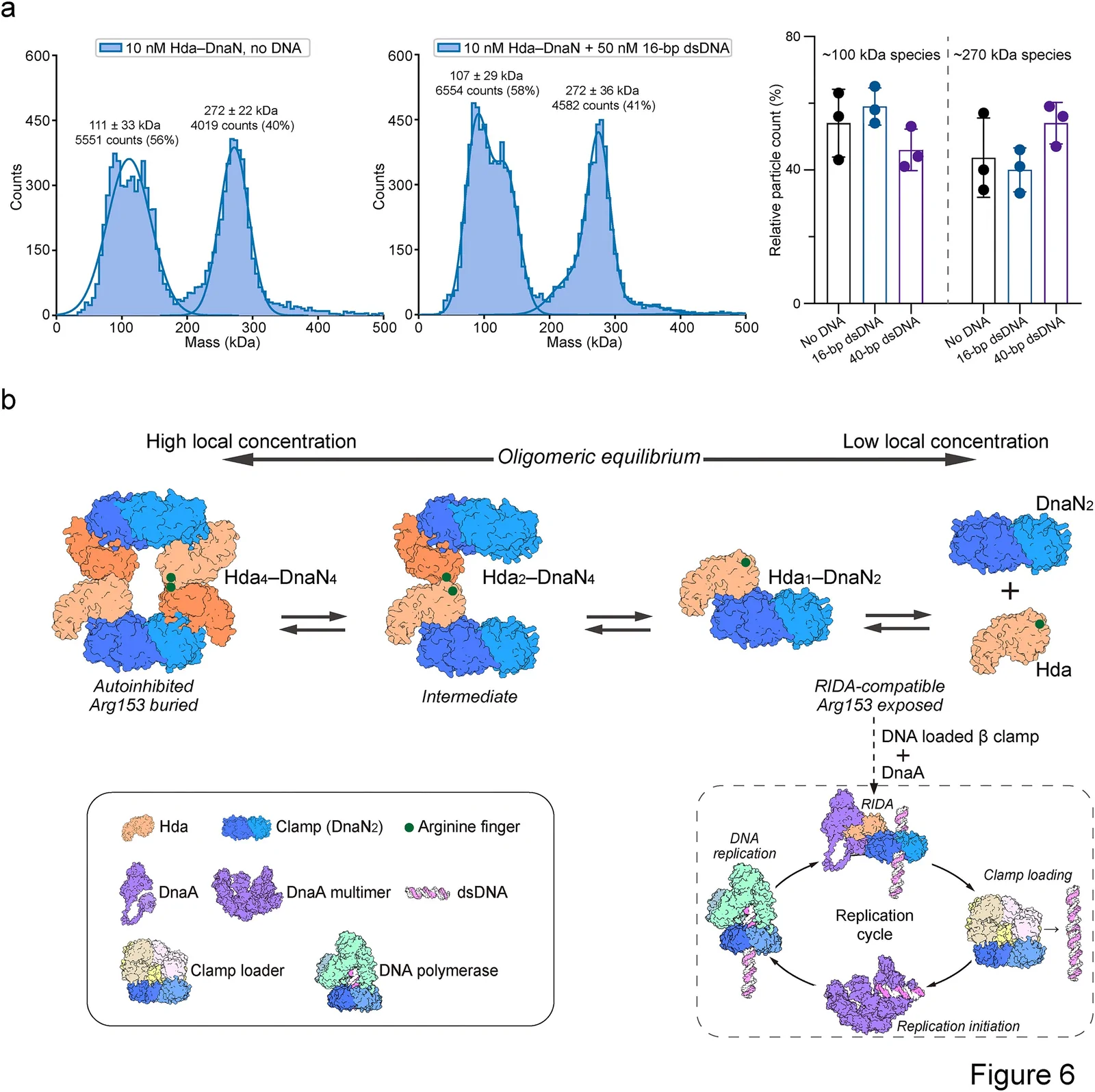
Effect of duplex DNA on Hda–DnaN oligomerization and proposed structural framework for RIDA regulation. (**A**) MP analysis of the Hda–DnaN complex in the absence or presence of duplex DNA. Representative mass distributions of 10 nM Hda–DnaN without DNA and in the presence of 50 nM 16-bp dsDNA are shown. The relative particle counts of the major ∼100- and ∼270-kDa species were quantified from three replicate measurements in the absence of DNA or in the presence of 16- or 40-bp dsDNA. Addition of dsDNA did not produce a consistent redistribution of the major Hda–DnaN oligomeric populations under the conditions tested. Complete MP distributions from all replicate measurements are shown in Supplementary Fig. S5. (**B**) Proposed structural framework for concentration-dependent Hda–DnaN oligomerization and RIDA regulation. Hda–DnaN populates multiple oligomeric states whose relative abundance varies with protein concentration *in vitro*. The resolved Hda_4_–DnaN_4_, Hda_2_–DnaN_4_, and Hda_1_–DnaN_2_ structures are depicted as interconverting states within this oligomeric landscape. In the higher-order Hda_4_–DnaN_4_ assembly, the Arg153-containing surface of Hda is buried at the Hda–Hda interface, whereas lower-order Hda_1_–DnaN_2_ assemblies expose this region and are structurally compatible with DnaA engagement. Physiological RIDA occurs in the context of DNA-loaded DnaN; previous biochemical and genetic studies support the physiological relevance of Hda–DnaN association, although the extent to which individual higher-order assemblies are populated *in vivo* and how replication-associated factors influence this equilibrium remain to be determined.

In the absence of DNA, the two major populations were centered at ~100 and 270 kDa. Addition of 16-bp dsDNA produced a modest redistribution toward the ∼100-kDa population, with a corresponding decrease in the ∼270-kDa population. However, this tendency varied among replicate measurements and was not reproduced with 40-bp dsDNA. Instead, the 40-bp condition showed a modest shift in the opposite direction, with a slightly increased proportion of the ∼270-kDa population (Fig. 6a and Supplementary Fig. S5). Given the variability among replicate measurements and the absence of a consistent response across the two DNA lengths, we do not interpret these modest differences as evidence for a DNA-dependent change in Hda–DnaN oligomerization.

Thus, under the conditions tested, free dsDNA alone did not produce a consistent redistribution of the major Hda–DnaN oligomeric populations. Complete MP distributions from all replicate measurements, including mirrored background events, are shown in Supplementary Fig. S5.

## Discussion

Our structural and biophysical analyses support a model in which the Hda–DnaN complex populates multiple oligomeric states. Cryo-EM reconstruction resolved Hda_4_–DnaN_4_, Hda_2_–DnaN_4_, Hda_1_–DnaN_2_, and DnaN_2_ assemblies, whereas MP revealed a concentration-dependent redistribution between higher- and lower-molecular-weight species. Together, these observations indicate that Hda–DnaN possesses an intrinsic oligomeric equilibrium rather than existing as a single, fixed assembly. Structural comparison among the resolved states suggests a plausible route of interconversion. Relative to Hda_4_–DnaN_4_, the Hda_2_–DnaN_4_ intermediate lacks one Hda dimer and displays pronounced remodeling of the remaining Hda–Hda interface. Further disruption of this interface would generate lower-order Hda_1_–DnaN_2_ assemblies in which the Arg153-containing surface is exposed and structurally accessible for interaction with DnaA. We therefore interpret the resolved structures as representative states within a concentration-dependent oligomeric landscape rather than as evidence for an obligatory physiological sequence beginning from the Hda_4_–DnaN_4_ complex *in vivo*.

Building upon the static hetero-octamer crystal structure and biochemical analyses reported by Kim *et al*. [20], our results provide a structural view of the oligomeric plasticity of the Hda–DnaN complex. While the crystal structure revealed an autoinhibited assembly with a buried arginine finger, it did not explain how a RIDA-compatible Hda state could become accessible. Here, our cryo-EM structures extend this model by revealing multiple Hda–DnaN oligomeric states, including an intermediate Hda_2_–DnaN_4_ assembly and a lower-order Hda_1_–DnaN_2_ complex in which the arginine finger is exposed. Together with the concentration-dependent redistribution observed by MP, these structures support a model in which Hda–DnaN samples an oligomeric landscape encompassing autoinhibited and RIDA-compatible states.

Structural and MD analyses indicate that this transition is primarily driven by progressive destabilization of the Hda–Hda (HH) interface, whereas the Hda–DnaN (HD) interaction remains largely preserved (Figs 2–5). This asymmetric stability provides a structural basis for a possible route of oligomeric interconversion in which disruption of Hda self-association could precede dissociation from DnaN. These findings suggest that the Hda–DnaN complex has an intrinsic capacity for reversible oligomerization, potentially modulating the accessibility of Hda surfaces involved in DnaA regulation. Notably, Hda occupies the front face of DnaN, similar to many canonical clamp-binding proteins, including replicative DNA polymerases. This shared binding surface suggests that Hda recruitment is integrated with the broader network of DnaN partner interactions and may require coordinated exchange with other clamp-associated factors during replication. Thus, accessibility of the DnaN front face may provide an additional layer of regulation linking Hda recruitment to the replication status of DnaN.

In addition, AlphaFold-based modeling provides a hypothesis for how the active Hda_1_–DnaN_2_ complex engages DnaA. The predicted model suggests that upon DnaA binding, the Hda lid and base domains undergo a large conformational rearrangement, while maintaining interaction with DnaN, thereby exposing interaction surfaces that contact multiple domains of DnaA (Supplementary Fig. S6). Given the moderate ipTM score (0.60), this model should be regarded as a hypothesis for possible DnaA engagement rather than a definitive representation of the RIDA complex. Although this model requires experimental validation, it offers a plausible structural framework for understanding how Hda coordinates ATP hydrolysis, disruption of DnaA oligomerization, and inhibition of DNA binding.

Building on these findings, we propose a concentration-dependent oligomeric model for the Hda–DnaN complex (Fig. 6b). Our cryo-EM structures reveal Hda_4_–DnaN_4_, Hda_2_–DnaN_4_, and Hda_1_–DnaN_2_ assemblies, while MP shows that the relative abundance of higher- and lower-order species varies with protein concentration. We therefore interpret these structures as states within an oligomeric landscape rather than as an obligatory sequential pathway *in vivo*. Higher-order assemblies are favored at higher concentrations, whereas lower-order species become enriched upon dilution. Among these states, the Hda_1_–DnaN_2_ complex exposes the Arg153-containing surface and is structurally compatible with DnaA engagement. How these oligomeric states are populated and interconverted in the physiological context of DNA-loaded β clamps remains to be established.

Although the abundance and lifetime of individual Hda–DnaN oligomeric states *in vivo* remain unknown, previous quantitative and biochemical studies support the physiological plausibility of Hda–DnaN association at concentrations relevant to the equilibrium observed here. Su’etsugu *et al*. estimated ~50 Hda dimers (∼100 Hda monomers) per *E. coli* cell [38], while previous estimates cited by Kim *et al*. place the cellular abundance of DnaN at ~250–500 dimers per cell [20]. Consistent with these cellular abundance estimates, quantitative ribosome profiling by Li *et al*. reported synthesis rates of ~99–188 Hda molecules and 529–1764 DnaN molecules per generation, depending on the growth condition [39]. For a typical *E. coli* cell volume of ~1–2 fL, an abundance of ∼100 Hda monomers per cell corresponds to a whole-cell average concentration of ~80–170 nM. Thus, although cellular abundance cannot be directly equated with the concentration of individual Hda–DnaN oligomeric states, these estimates place the physiological abundance of Hda within a concentration regime relevant to the nanomolar range examined in our MP experiments.

Previous biochemical studies further showed that the cellular abundance of DnaN substantially exceeds that of Hda and suggested that a substantial fraction of Hda may therefore be associated with DnaN *in vivo*. Importantly, Kawakami *et al*. demonstrated that a preformed Hda–DnaN complex remains competent for DnaA–ATP hydrolysis and that its DnaN component can be loaded onto DNA by clamp-deficient pol III [40], indicating that Hda association with DNA-unloaded DnaN can precede clamp loading without abolishing Hda function. Together with the interface mutational analyses of Kim *et al*., which showed that perturbation of the Hda–Hda interface affects both higher-order assembly and RIDA activity, these observations support the potential physiological relevance of Hda–DnaN oligomerization. Nevertheless, direct determination of the oligomeric distribution of Hda–DnaN in living cells will be required to establish the extent to which the higher-order assemblies identified here are populated *in vivo*.

Our additional MP measurements further indicate that free dsDNA alone is not sufficient to impose a consistent shift in the Hda–DnaN oligomeric distribution under the conditions tested (Fig. 6a and Supplementary Fig. S5). Although the 16-bp duplex produced a modest enrichment of the ∼100-kDa population, this effect was variable among replicate measurements and was not reproduced with the longer 40-bp duplex, which showed a small shift in the opposite direction. We therefore consider these differences to reflect experimental variability rather than evidence for a DNA-length-dependent effect on Hda–DnaN assembly.

Importantly, these experiments examine the effect of free dsDNA and do not reproduce the topological state of DnaN loaded onto DNA. *In vivo*, DnaN is opened and loaded around primer–template DNA by the clamp-loader complex, after which the loader dissociates, and the closed clamp remains topologically linked to DNA [41]. Recent structural analyses of the complete *E. coli* clamp-loading cycle have directly visualized this process and the conformational rearrangements accompanying DNA loading [15]. Thus, the absence of a consistent effect of free dsDNA in our measurements does not exclude a role for DNA in physiological RIDA. Rather, the topological loading of DnaN onto DNA, together with other replication-associated factors, may provide a distinct molecular environment that influences Hda–DnaN organization. Direct examination of Hda assembly on DNA-loaded DnaN will be required to address this possibility.

This model is consistent with previous structural and functional analyses of the Hda–DnaN complex [1, 2, 18–20]. The crystal structure showed that Hda–Hda interactions occlude the catalytic arginine finger required for DnaA ATP hydrolysis, and mutational studies demonstrated that perturbing Hda–Hda or Hda–DnaN interfaces disrupts RIDA function. Our findings extend this framework by revealing additional Hda–DnaN oligomeric states that connect structurally autoinhibited and RIDA-compatible configurations.

More broadly, our results place Hda–DnaN assembly dynamics within the regulatory network controlling bacterial DNA replication. In *E. coli*, replication initiation depends on the balance between ATP–DnaA and ADP–DnaA [5–7, 19], which is modulated by multiple systems including RIDA, datA-dependent hydrolysis, and DARS-mediated nucleotide exchange [42]. This ATP–DnaA/ADP–DnaA cycle functions as a molecular timer that coordinates replication initiation with the cell cycle [5, 42, 43].

In this context, Hda–DnaN oligomerization may provide an additional layer of regulation by modulating the accessibility of functionally important Hda surfaces. Structurally, the Hda_4_–DnaN_4_ hetero-octamer represents an autoinhibited state in which the arginine finger is occluded, whereas the Hda_1_–DnaN_2_ complex exposes this region and is therefore compatible with DnaA engagement. Such oligomeric plasticity could allow Hda availability to respond to local concentration, DnaN availability, and replication-coupled recruitment.

Importantly, this behavior could be understood in the framework of the classical titration model of replication control. In *E. coli*, DnaA activity is regulated not only through ATP hydrolysis but also through titration onto chromosomal binding sites and regulatory complexes [5]. Our results raise the possibility that the concentration-dependent oligomeric behavior of Hda–DnaN could provide a titration-like mechanism for modulating the availability of RIDA-compatible Hda. The concentration dependence observed *in vitro* provides a potential mechanism by which the oligomeric state of Hda–DnaN could respond to changes in its local molecular environment. At higher concentrations, higher-order assemblies are favored, whereas dilution shifts the distribution toward lower-order species. Because these states differ in the accessibility of the Hda arginine-finger region, such an equilibrium could, in principle, modulate the availability of RIDA-compatible Hda. Whether this concentration-dependent behavior contributes to RIDA regulation *in vivo*, however, remains to be determined.

Our results also highlight the power of combining cryo-EM with MP for studying dynamic protein assemblies. SEC alone failed to resolve distinct Hda–DnaN species detected by MP, potentially reflecting their overlapping chromatographic behavior and/or exchange between oligomeric states. In contrast, MP directly detected multiple coexisting species in solution, while cryo-EM provided structural snapshots of these assemblies. This integrative approach is particularly well-suited for systems that are heterogeneous, oligomeric, or in dynamic equilibrium.

An important methodological consideration is whether transitions between different oligomeric states can be directly observed by MP. In principle, MP offers sufficient temporal resolution to detect changes in molecular weight upon particle landing. However, in practice, it captures stochastic surface-binding events of individual particles rather than continuous trajectories of the same molecule. As a result, for heterogeneous systems such as Hda–DnaN, the measurements primarily reflect the distribution of species present under the measurement conditions rather than real-time interconversion between individual states.

Consistent with this, we did not directly observe discrete conversion events between hetero-octameric and hetero-hexameric states during individual recordings. Instead, the presence of multiple coexisting populations and their concentration-dependent redistribution supports the existence of a concentration-dependent oligomeric equilibrium. This interpretation is consistent with previous applications of MP to dynamic protein assemblies, where heterogeneous populations rather than transition trajectories are typically resolved.

Several limitations remain. First, our experiments do not establish whether the Hda_4_–DnaN_4_ complex accumulates appreciably *in vivo* or whether it represents an obligatory precursor of the RIDA-active state. Second, the directionality and kinetics of assembly–disassembly transitions cannot be directly inferred from static cryo-EM structures. Third, the AlphaFold-based RIDA complex model remains predictive and requires experimental validation. Finally, although free dsDNA did not consistently alter Hda–DnaN oligomerization in our MP experiments, the effects of topological DnaN loading onto DNA and other replication-associated factors remain to be defined.

In conclusion, our study reveals a concentration-dependent oligomeric landscape of the Hda–DnaN complex. By defining multiple oligomeric states spanning autoinhibited and RIDA-compatible configurations, we provide a structural framework for understanding how Hda–DnaN oligomerization may contribute to the regulation of bacterial replication licensing.

## Supplementary Material

gkag1010_Supplemental_Files

## Data Availability

All data are provided in the main text and the Supplementary Information. The structural models have been deposited in the Protein Data Bank (PDB) under accession codes 25RY (Hda_4_–DnaN_4_ hetero-octamer), 25RZ (Hda_2_–DnaN_4_ hetero-hexamer), 25SA (Hda_1_–DnaN_2_ hetero-trimer), and 25WZ (DnaN_2_ homodimer). The corresponding cryo-EM maps, along with half-maps, FSC curves, and masks, have been deposited in the Electron Microscopy Data Bank (EMDB) under accession codes EMD-80334 (Hda_4_–DnaN_4_), EMD-80335 (Hda_2_–DnaN_4_), EMD-80336 (Hda_1_–DnaN_2_), and EMD-80433 (DnaN_2_).
