# Supplementary material for: Reversible autoinhibition of the bacterial replication-licensing Hda–β clamp complex
Source: Nucleic Acids Res. 2026 Oct 23;54(19):gkag1010. doi: 10.1093/nar/gkag1010 (PMC13691371; doi:10.1093/nar/gkag1010)
Supplement: Supplementary file 1 [file gkag1010_supplemental_files.zip › movie 1 legends.docx]

**VIDEO**

**Movie 1. Molecular dynamics simulation of the Hda_2_–DnaN_4_ complex.**

The trajectory shows structural changes of the Hda_2_–DnaN_4_ complex during a 1 ns molecular dynamics simulation, starting from a model generated by removing one Hda dimer from the Hda_4_–DnaN_4_ assembly. A total of 100 frames (0–1 ns) are displayed as a backbone ribbon representation, illustrating the structural relaxation of the intermediate state.
