# Supplementary material for: Reversible autoinhibition of the bacterial replication-licensing Hda–β clamp complex
Source: Nucleic Acids Res. 2026 Oct 23;54(19):gkag1010. doi: 10.1093/nar/gkag1010 (PMC13691371; doi:10.1093/nar/gkag1010)
Supplement: Supplementary file 1 [file gkag1010_supplemental_files.zip › SI2609.docx]

Supplementary Information for

**Reversible autoinhibition of the bacterial replication-licensing Hda–β clamp complex**

Xuguang Jiang *et al.*

*Masahide Kikkawa. Email: mkikkawa@m.u-tokyo.ac.jp

**This PDF file includes:**

Supplementary Figs. 1 to 6

Supplementary Fig. 1.

**(a)** Theoretical molecular weights of Hda, DnaN, and their complexes.

**(b)** Mass photometry analysis of independently purified His-tagged DnaN fractions following size-exclusion chromatography.

**Supplementary Fig. 2. Complete mass photometry distributions of the Hda–DnaN complex at different concentrations.**

Complete mass photometry distributions corresponding to the concentration-dependent measurements shown in Fig. 1e. Hda–DnaN was analyzed at the indicated concentrations ranging from 0.625 to 20 nM. Molecular masses are shown as mean ± SD of the fitted distributions, together with particle counts and relative fractions for the major populations. Negative-mass events represent mirrored background landing events and are displayed to facilitate evaluation of low-molecular-weight signals.

Supplementary Fig. 3. Cryo-EM data processing workflow for the Hda–DnaN complex.

Supplementary Fig. 4. Structural alignment of trimeric and dimeric subunits within different Hda–DnaN assemblies.

**(a)** Alignment of the two heterotrimeric units within the Hda_2_–DnaN_4_ hetero-hexamer.

**(b)** Alignment of the trimeric units between the Hda_1_–DnaN_2_ and Hda_4_–DnaN_4_ assemblies.

**(c)** Structural alignment between the Hda_1_–DnaN_2_ complex and the DnaN dimer (DnaN_2_).

**Supplementary Fig. 5. Mass photometry analysis of Hda–DnaN in the presence of duplex DNA.**

Complete mass photometry distributions from three replicate measurements of 10 nM Hda–DnaN in the absence of DNA (upper), in the presence of 50 nM 16-bp dsDNA (middle), or in the presence of 50 nM 40-bp dsDNA (bottom). Molecular masses are shown as mean ± SD of the fitted distributions, together with particle counts and relative fractions for the major populations. Negative-mass events represent mirrored background landing events and are displayed to facilitate evaluation of low-molecular-weight signals. Quantification of the major ~100-kDa and ~270-kDa populations is shown in Fig. 6a.

Supplementary Fig. 6. AlphaFold-predicted model of the DnaA–Hda–DnaN_2_ complex.

(**a**) AlphaFold 3 prediction of the DnaA–Hda–DnaN_2_ complex. In the right panel, the residue order corresponds to Hda, two DnaN subunits, and DnaA.

(**b**) Structural representation of the predicted complex. The 2D schematic illustrates domain organization and predicted intermolecular domain interactions. The left panel highlights conformational differences of Hda relative to the cryo-EM Hda_1_–DnaN_2_ structure.
